# Mixed-methods, participatory action research study exploring palliative and end-of-life care for LGBTIQ+ people in Switzerland: a protocol for the TRUST-PALL study

**DOI:** 10.1136/bmjopen-2025-106641

**Published:** 2026-01-07

**Authors:** Michael J Deml, Clément Meier, Catarina Letras, Nina Canova, Paolo Martinelli, Laura Jones, Gaé Colussi, Philip J Larkin, Léïla Eisner, Tabea Hässler, Francesca Bosisio, Claudia Gamondi

**Affiliations:** 1 Palliative and Supportive Care Service, Lausanne University Hospital (CHUV), Lausanne, Switzerland; 2 Institute of Sociological Research, University of Geneva, Geneva, Switzerland; 3 Faculty of Business and Economics (HEC), University of Lausanne, Lausanne, Switzerland; 4 Swiss Centre of Expertise in the Social Sciences (FORS), Lausanne, Switzerland; 5 School of Management and Engineering Vaud (HEIG-VD), HES-SO University of Applied Sciences and Arts of Western Switzerland, Yverdon-les-Bains, Switzerland; 6 Institute of Higher Education and Research in Healthcare, Faculty of Biology and Medicine, University of Lausanne, Lausanne, Switzerland; 7 University of Lausanne Institute of Psychology, Lausanne, Switzerland; 8 Department of Psychology, University of Zurich, Zürich, Switzerland

**Keywords:** PALLIATIVE CARE, Sexual and Gender Minorities, Health Equity, Adult palliative care, Patient Participation, STATISTICS & RESEARCH METHODS

## Abstract

**Abstract:**

**Introduction:**

Lesbian, gay, bisexual, trans, intersex, queer/questioning and other sexual and gender minorities (LGBTIQ+) face systemic barriers and discrimination in healthcare settings, leading to significant health disparities. These challenges persist in palliative and end-of-life care (PEOLC), where older LGBTIQ+ people often lack family support and experience social isolation. Despite the increasing ageing of the LGBTIQ+ population in Switzerland, there is limited evidence on their specific PEOLC needs. Additionally, healthcare providers’ knowledge and practices regarding LGBTIQ+ inclusivity in these settings remain understudied. This study aims to address these gaps by co-creating knowledge and developing best practice recommendations for inclusive PEOLC in Switzerland.

**Methods and analysis:**

This study employs a mixed-methods participatory action research approach across three work packages (WPs). WP0 ensures participatory engagement through advisory boards, workshops and co-design processes across Switzerland’s four linguistic regions. WP1 investigates the palliative and PEOLC needs of LGBTIQ+ people and their (chosen) families through qualitative interviews (n≈30) and a quantitative survey embedded in the Swiss LGBTIQ+ Panel. WP2 explores healthcare providers’ perceptions and practices regarding LGBTIQ+ patients through qualitative interviews (n≈30) and a nationwide quantitative survey among palliative and PEOLC professionals. Data will be analysed using reflexive thematic analysis for qualitative data and multivariate regression models for quantitative data. Findings will be synthesised through a specific data integration framework, integrating community and healthcare perspectives.

**Ethics and dissemination:**

This study has received ethical approval from the relevant Swiss Ethics Committees. The participatory approach promotes inclusivity, empowering LGBTIQ+ people and healthcare providers in shaping recommendations. Findings will be disseminated via peer-reviewed publications, policy briefs, stakeholder workshops and the co-created Rainbow Book, a best-practice guide for LGBTIQ+ inclusive palliative and PEOLC in Switzerland.

**STRENGTHS AND LIMITATIONS OF THIS STUDY**

- This study uses a participatory action research approach that actively involves lesbian, gay, bisexual, trans, intersex, queer/questioning and other sexual and gender minorities (LGBTIQ+) people, their (chosen) families and healthcare providers throughout all research stages.
- The mixed-methods design combines qualitative interviews, secondary analyses of national datasets and a large-scale survey to provide a comprehensive understanding of LGBTIQ+ palliative and end-of-life care needs.
- Recruitment of marginalised and older LGBTIQ+ individuals may be challenging, which could result in limited participation from people less connected to community networks.
- Coordinating participatory processes across multiple linguistic regions may introduce methodological complexity despite strategies to support multilingual engagement.
- The integration of qualitative and quantitative findings requires careful methodological alignment, which may limit the depth of analyses within individual components.

## Introduction

 Lesbian, gay, bisexual, trans, intersex, queer/questioning and other sexual and gender minorities (LGBTIQ+) often face unique challenges in healthcare settings, including palliative and end-of-life care (PEOLC). The experience of *minority stressors*,^1^ including structural barriers to access care, discrimination, inequalities and stigma, may lead to mistrust of the health system. These stressors extend to *families of origin* (ie, the parents, siblings or extended kin with whom one was raised) and *families of choice* (ie, loving, caring and supportive relationships that LGBTIQ+ people build with peers and friends beyond families of origin). Minority stressors, such as structural inequalities, marginalisation and discrimination of LGBTIQ+ people persist in wider society and healthcare settings,^2 3^ leading to health disparities at all stages of life.^4^,^7^

Based on European data, we estimate that 10% of the Swiss population belongs to a sexual and/or gender minority.^8^ Recent large-scale studies, such as the LGBT Health Survey^3^ and the Swiss LGBTIQ+ Panel,^2^ have documented significant health disparities between LGBTIQ+ and cisgender endosex heterosexual people (ie, heterosexual people whose gender identity matches their assigned sex at birth and whose sex characteristics fit normative medical and social ideas for female or male bodies), including well-being, mental health, substance use, sexual and physical health. Health disparities are particularly pronounced among gender minority members, making them a particularly vulnerable group within the LGBTIQ+ community.^3^ In the 2021 Swiss LGBT Health Survey, a majority of the 2064 respondents reported carefully negotiating their coming out, encountering discrimination or violence based on their sexual orientation and/or gender identity, with 26.6% reporting such incidents within healthcare settings.^2^

LGBTIQ+ populations encompass heterogeneous subgroups, diverse in terms of gender, sexuality, sex characteristics, age, migration status, ethnicity, religion, ability, socioeconomic status, relationship and family status. Research needs to ask questions that effectively disentangle the nuances in these inequalities. Not doing so results in sections of the LGBTIQ+ community being misrepresented, conflated with other groups or erased altogether.^9^,^11^ Previous negative interactions with healthcare throughout LGBTIQ+ people’s lives may result in feelings of distrust, eliciting reluctance to seek care.^12^,^14^ Many LGBTIQ+ people grew up at a time when sexual orientation, gender identity and/or intersex status were widely stigmatised, pathologised or even criminalised,^15^ leading to chronic adversity and enduring silence. In addition, older LGBTIQ+ people are more inclined to live alone, which increases their vulnerability to social isolation.^9 16 17^ The Swiss LGBTIQ+ population aged 65 and older is expected to double and reach 270 000 by 2045,^8^ and tailored interventions and improvements in healthcare equity for the older LGBTIQ+ community, including awareness and preparedness of healthcare professionals, are needed.

The majority of deaths in Switzerland, as in other high-income countries, occur among the older segment of the population. The primary causes of death include cardiovascular diseases, cancer, respiratory diseases, accidents, acts of violence and dementia.^18^ In 2023, Switzerland recorded 71 822 deaths, with 24.7% involving people aged 65–79 and 63.7% involving those aged 80 or older.^19^ These deaths typically result from the gradual progression of diseases and declining functional abilities over the last years of life.^20^ As a result, older adults often undergo one or more hospitalisations in their final phase of life,^21 22^ necessitating critical medical decisions.^23^ End-of-life healthcare decisions often involve managing pain and symptoms, choosing whether to withhold or discontinue life-support treatments such as cardiopulmonary resuscitation, artificial nutrition and hydration, mechanical ventilation or dialysis, and, in Switzerland, considering the option of assisted dying.^24 25^ These decisions are typically made collaboratively by cognitively capable patients, informal caregivers and healthcare providers (HCPs).^26^,^28^ Factors influencing these decisions include patient prognosis, potential benefits and risks of treatments, and personal values and care goals.^24^ This complex decision-making process is often made in emotionally charged situations with complex family dynamics involving patients, their loved ones or designated healthcare proxies, and HCPs.^29^ Other personal factors, such as perceived meaning in life, which often relate to family, social relationships, spirituality, religion, social commitment and personal growth, also play important roles in end-of-life decisions.^30^

While end-of-life needs and decision-making processes for the general older population are well documented in the literature, there is limited evidence addressing the needs of LGBTIQ+ populations and their (chosen) families when confronted with life-threatening illnesses and the final stages of their lives. A systematic review reported that most research outside of Switzerland has focused on cancer experiences of gay men and lesbian women, with little mention of bisexual and pansexual people, and no evidence published for trans, non-binary and intersex people.^14^ Inclusion of other sub-populations within the LGBTIQ+ community (ie, intersectionality) in research is increasing, although this remains insufficient.^15^ Furthermore, LGBTIQ+ people’s support systems likely differ from cis-heteronormative family structures, which tend to rely on families of origin for support and care as they age or face illness. LGBTIQ+ people may rely more on families of choice, including friends and (ex-)partners,^31^ which has implications for healthcare professionals and health services, for example, in exploring social networks and possible healthcare proxies during Advance Care Planning discussions.^32^ International literature has highlighted the importance of social support and inclusion of (chosen) families in health and end-of-life decisions, fears of discrimination and lack of trust in healthcare institutions.^14 33^ These reviews also identified low knowledge and preparedness for end-of-life contexts and needs for increased economic and legal support in navigating healthcare systems.^14 33^ LGBTIQ+ patients in palliative situations may experience potential loss or erosion of identity, fear of rejection, neglect or disrespect by care staff.^34 35^ Although there is a growing body of international research on the LGBTIQ+ population, little to no evidence exists about Swiss LGBTIQ+ patients’ preferences in relation to healthcare needs and psychosocial support in palliative and end-of-life contexts of care in relation to age, type of illness and LGBTIQ+ subgroup, and national best practice recommendations are lacking.

Furthermore, there is limited evidence about the experience, perceptions and training of PEOLC professionals regarding their provision of care to LGBTIQ+ people in Switzerland. In studies outside of Switzerland, hospice care workers tend to consider sexual and gender minority status as being private, irrelevant for care provision and not something for which PEOLC needs to be tailored.^36^ In one survey, palliative care professionals in the USA reported that patients belonging to sexual and/or gender minorities, especially trans patients, were more likely to be subjected to discriminatory care in their institutions.^37^ Small-scale, exploratory studies in Switzerland have echoed these findings from perspectives of community home care providers,^38^ elderly long-term facilities^39^ and palliative care settings.^40^ These limited, non-representative Swiss findings suggest low awareness of LGBTIQ+-specific PEOLC needs, particularly for trans, gender diverse and intersex people,^39^ and indicate that professionals may not see a need for action.^39 40^ This stands in stark contrast to a Swiss report summarising surveys administered to LGBTIQ+ people, indicating their concerns that professionals would have low knowledge, preparation, openness, acceptance and training around LGBTIQ+-specific issues in home care, hospital and clinical settings and elderly long-term facilities.^41^ Despite consensus about training needs in this area, proper training and consistent, standardised competencies for appropriate and respectful care have not been developed.^42^

Switzerland lacks information about the specific needs of LGBTIQ+ people, their (chosen) families and HCPs in life-threatening illness and end-of-life care contexts. To address these gaps, we will employ a participatory action research (PAR) approach to co-create new knowledge on inclusive and respectful PEOLC for LGBTIQ+ people in Switzerland’s four linguistic regions. Our team of interdisciplinary researchers, specialised in PAR, PEOLC, psychology, sociology, public health and gender studies, has worked together to prepare the study we refer to as TRUST-PALL: Tailoring Respectful and Understanding SupporT for LGBTIQ+ individuals in PALLiative and end-of-life contexts. Our approach will bridge scientific and experiential knowledge to develop socially relevant healthcare interventions that address inequalities. The study has the following four objectives:

*Objective 1*: to investigate shared and distinct needs in PEOLC among LGBTIQ+ subpopulations, considering factors such as sex, gender, sexual orientation, intersex status, country of origin, migratory status, educational attainment, socioeconomic status, religion, disability and health status.

*Objective 2*: to identify how LGBTIQ+ people and their (chosen) families access palliative care and prepare for end-of-life care, including facilitators, barriers and preferred pathways of care.

*Objective 3*: to explore HCPs’ perceptions and understanding of LGBTIQ+ people in PEOLC, considering factors such as professional role, training, institutional support, ethical and legal considerations, workplace culture, personal beliefs and cultural humility.

*Objective 4*: to identify how HCPs deliver PEOLC to LGBTIQ+ people, including facilitators and barriers to creating inclusive and sensitive care environments.

## Methods and analysis

**Figure 1:**
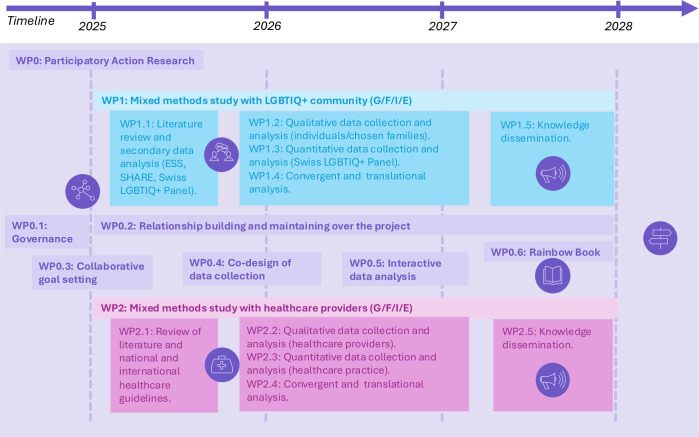
Tailoring Respectful and Understanding SupporT for LGBTIQ+ individuals in PALLiative and end-of-life contexts methodological design. The colours were chosen to represent key themes: blue for trust, purple for palliative care, and their combination, indigo, to symbolise the integration of these values through the participatory action research approach central to the project. LGBTIQ+, lesbian, gay, bisexual, trans, intersex, queer/questioning and other sexual and gender minorities; SHARE, Survey of Health, Ageing and Retirement in Europe; WP, work package; ESS, Swiss Health Survey.

Our research adopts a PAR approach, ensuring that the voices and experiences of LGBTIQ+ people, their (chosen) families and HCPs emerge and are used in the development of interventions and recommendations. This approach is embedded within a convergent mixed-methods experimental design (figure 1), in which qualitative and quantitative data are collected in parallel, analysed separately and then integrated to provide a comprehensive understanding of the PEOLC experiences of LGBTIQ+ people and HCPs.^14^ The project is structured into three distinct work packages (WPs):

*WP0*: this WP oversees the *PAR framework*, ensuring that the methodologies employed in each subsequent WP align with PAR principles.

*WP1*: this mixed-methods study focuses on the *Swiss LGBTIQ+ community*, addressing objectives 1 and 2.

*WP2*: this mixed-methods study targets *HCPs*, focusing on objectives 3 and 4.

All WPs are integrated into our PAR approach WP0. WP1 addresses our first two objectives and consists of a qualitative study component with LGBTIQ+ people and their (chosen) families, secondary data analysis of pre-existing quantitative datasets, and a quantitative study component with the Swiss LGBTIQ+ Panel. WP2 addresses objectives 3 and 4 through qualitative and quantitative study components with HCPs. All participants will provide informed consent prior to participation. Adult participants (aged 18 and older) will provide written consent for qualitative interviews and electronic consent for survey participation. For participants aged 14–17, consent will be obtained directly from the adolescent, in line with Swiss ethical standards for minimal-risk social science research. In accordance with the ethical approval procedures of the Swiss LGBTIQ+ Panel, parental or legal guardian consent is not required, as this could compromise the safety and confidentiality of minors in sensitive identity-related research. Also, no financial incentives will be offered to participants. For qualitative interviews, participants will be reimbursed for travel expenses where applicable, and refreshments will be provided during workshops to acknowledge their time and facilitate participation.

## WP0—participatory action research

PAR is a collaborative and participatory research approach that involves repetitive cycles of research, action and reflection to continuously refine and adapt the study as it progresses. It highlights the importance of involving the people affected by the research to bridge scientific and experiential knowledge. PAR emphasises the importance of amplifying marginalised voices in the knowledge creation process to empower communities and drive social change. For it to be successful, it must involve working with and alongside the recipients of the project, rather than conducting research on them.^43^,^46^ PAR is used in healthcare to improve the relevance of healthcare services and outcomes from the point of view of patients and their relatives and more broadly, empower them by increasing their healthcare literacy and decisional autonomy. It ensures that our study effectively tackles social and health disparities, strengthens cultural relevance and boosts the adoption of the co-developed recommendations, which shall be contextually appropriate, flexible and applicable.^47 48^

In this project, PAR serves as a foundational methodology that addresses the unique challenges faced by LGBTIQ+ people and ensures that the research outcomes are both socially relevant and actionable. PAR operates on several key principles that align closely with the constructivist and transformative goal of this project.

### Collaboration and participation

PAR emphasises the active involvement of LGBTIQ+ communities and people, their (chosen) families and HCPs. This collaboration ensures that the research process is inclusive, with community members contributing to develop or reframe research questions, data collection, analysis and dissemination of findings.

### Co-construction of knowledge

Recognising that knowledge is constructed through social interactions, PAR allows for the integration of diverse perspectives. This process acknowledges multiple realities and emphasises understanding the lived experiences of LGBTIQ+ people, their (chosen) families and HCPs within their specific sociocultural, situational and historical contexts.

### Reflexivity and positionality

Researchers in PAR engage in continuous reflexivity, acknowledging researchers’ own positionalities and biases. This reflexivity ensures that the research process remains transparent and that the voices and experiences of LGBTIQ+ community members are prioritised and authentically represented.

### Social justice and emancipatory goals

PAR is inherently oriented towards social justice, aiming to identify and address systemic inequities and barriers faced by LGBTIQ+ people in accessing respectful and inclusive PEOLC. The goal is to achieve emancipatory outcomes that transform healthcare practices to be more inclusive, respectful and responsive.

These four principles were selected as they represent the core foundations of PAR in health, emphasising co-learning, empowerment and social transformation.^43 47^ They align closely with the TRUST-PALL study’s goal to co-create inclusive and contextually grounded knowledge with LGBTIQ+ communities in sensitive palliative and end-of-life contexts. Based on these principles, WP0 in this project entails six steps: create a participative governance setting (WP0.1); build and sustain relationships over the project (WP0.2); collaborative goal setting (WP0.3); co-design of data collection (WP0.4); interactive data analysis (WP0.5); the Rainbow Book: conclusion and sustainability planning (WP0.6).

### WP0.1—create a participative governance setting

This initial phase involves engaging the study stakeholders: LGBTIQ+ community members, LGBTIQ+ associations, HCPs and other key social partners that provided support letters. Selected stakeholders will form a National Advisory Board (NAB) and three distinct operational boards to ensure cultural and linguistic representation. The NAB will play a key role in overseeing the research process and making sure it stays consistent with the project’s aims and considers local adaptations. The NAB will support the research team in the study process and provide feedback on data collection, analyses and findings. Moreover, the NAB will champion the recommendations of the study and by then support their implementation across cantons and languages.

**Figure 2:**
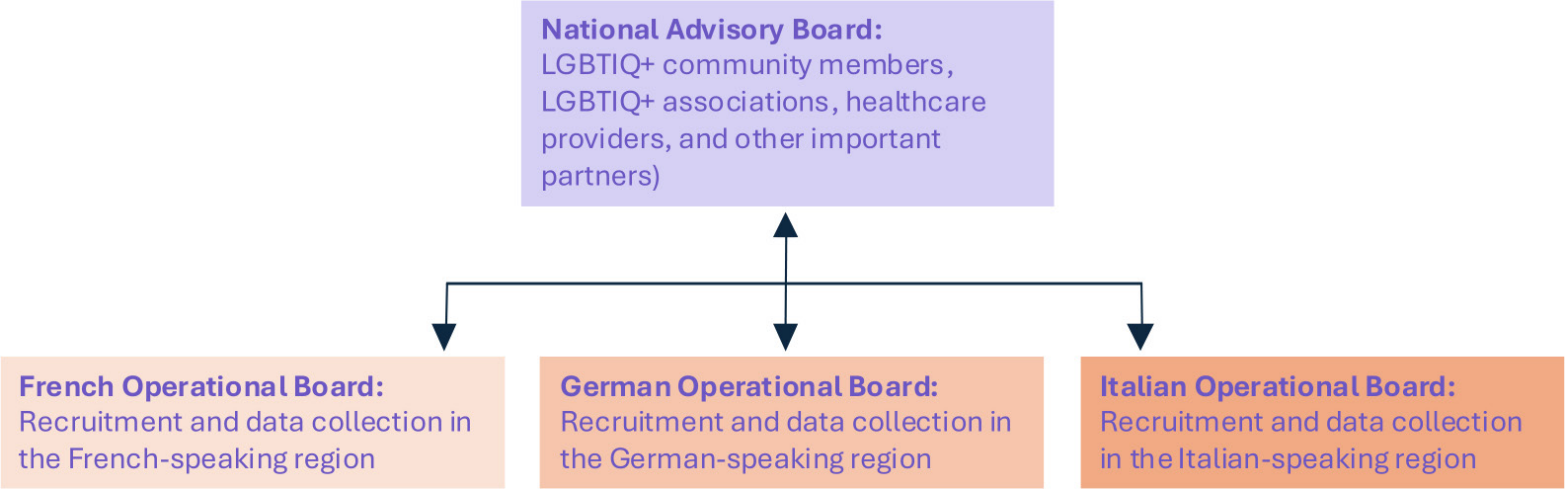
Illustration of the overall governance of the research. LGBTIQ+, lesbian, gay, bisexual, trans, intersex, queer/questioning and other sexual and gender minorities.

The regional operational boards will be responsible for coordinating research across Switzerland’s main linguistic regions, including participants from the Romansh region, a generally isolated part of Switzerland. Figure 2 illustrates the governance of the project and its function with the operational boards across linguistic regions. NAB meetings will be conducted in a federal language, meaning everyone can speak and answer in their preferred language while operational boards will be held in the three major regional languages (ie, German, French and Italian). This stage aligns with the principles of knowledge co-construction and contextual understanding by involving community members from the outset.

### WP0.2—building and sustaining relationships over the project

Throughout the research process, priority will be given to in-person workshops to foster relationship-building and trust. The composition of participants for each workshop will be discussed and determined in consultation with the NAB. Each workshop will start with an ice-breaker activity to facilitate introductions and discussion. A framework of ground rules will be established to ensure a safe and respectful environment. Special care will be taken to establish conditions that promote the participation of everyone, including the use of accessible language and a variety of formats in workshops and modes of expression. For participants uncomfortable with group settings, individual meetings may be arranged to incorporate their perspectives. At the end of each workshop, a synthesis document summarising key discussion points will be prepared by a team member and shared with all participants. Participants will review and provide feedback on the document to ensure that all relevant elements and points of view are accurately represented. *These activities support reflexivity and participant voices, ensuring that the research process respects and integrates the lived experiences of the participants*.

### WP0.3—collaborative goal setting

Prior to the data collection, three workshops (one in the German-, one in the French- and one in the Italian-speaking part) will be held on completion of the existing knowledge synthesis. Each workshop will bring together LGBTIQ+ people and their (chosen) families, LGBTIQ+ associations and HCPs. During these workshops, key elements identified in the knowledge synthesis for each WP will be presented, and participants will be asked to amend or validate the findings based on their experiences and knowledge. Since the literature review also includes international studies, participants will be asked to identify the elements they believe are relevant to the Swiss context. Furthermore, these workshops will provide an opportunity to enrich the synthesis with Swiss-specific insights that may not have been captured in the existing literature.

Participants will then collaboratively identify WP-relevant challenges and needs. Following a strengths-based approach,^49^ discussions will also focus on identifying the resources mobilised by the LGBTIQ+ community and people. Through a prioritisation activity inspired by the Agreement-&-Certainty Matrix methodology,^50^ participants will set research goals and objectives for the second phase of each WP (data collection and analysis). *This step models the transformative paradigm’s aim by addressing inequities and advocating for inclusive practices*.

### WP0.4—co-design of data collection

WP1 and WP2 data collection materials, such as interview or focus group guides and questionnaires, will be presented, and participants will be asked to assess their relevance and comprehensibility. The research team will collaborate with LGBTIQ+ communities involved in this project to recruit participants for WP1 (see WP1.1 and WP1.2) and involve community members as co-researchers for data collection. This will allow for the involvement of a bridge person^51^—someone who is both a researcher and member of the community—who can address any potential mistrust participants may have toward researchers or health professionals. *This stage supports the principles of multiple realities and contextual understanding by ensuring the data collection methods are designed with input from those who experience such issues firsthand*.

### WP0.5—interactive data analysis

Following data collection for each WP, three workshops will be organised to provide an opportunity to gather insights on primary findings, validate the data and collaboratively analyse the results, ensuring that diverse perspectives are considered when identifying themes and patterns. Finally, structured activities using Nominal Group Technique methods^52^ will be employed to reach a consensus on best practice recommendations. *This aligns with critical inquiry and emancipatory outcomes by involving participants in the analysis process to highlight power dynamics and ensure the findings lead to transformative changes*.

### WP0.6—The Rainbow Book: conclusion and sustainability planning

The project will conclude with a review of findings, acknowledgement of stakeholders, and planning for the sustainability of the project’s impacts. The ‘Rainbow Book’ is a novel output of the TRUST-PALL project. It symbolises inclusivity and diversity and will serve as a co-created best-practice guide translating project findings into accessible recommendations for LGBTIQ+ people, their (chosen) families, healthcare professionals and policymakers. To co-develop the Rainbow Book, two sets of workshops will be organised in each of the French-, German- and Italian-speaking parts of Switzerland. The book will be designed for specific audiences: (1) LGBTIQ+ organisations, (2) LGBTIQ+ patients and (chosen) families, (3) health professionals, with emphasis on expanding their cultural humility in care with LGBTIQ+ populations and (4) health authorities and policy makers.

The first set of workshops will involve LGBTIQ+ people and their (chosen) families, LGBTIQ+ associations and HCPs. Findings will be presented to participants, who will be divided into small groups of 4–6 participants to collaboratively define recommendations and possible next steps. A spokesperson from each group will present their recommendations to other groups, starting with the groups of LGBTIQ+ people and their (chosen) families. Those presentations will be followed by a discussion to reach a consensus on the key elements for each recommendation. After these workshops, LGBTIQ+ people and their (chosen) families will be invited to participate in a Photovoice activity.^53^ If they accept, they will receive training and be asked to take photos that represent their lived experiences in relation to challenges or resources underlying a best practice recommendation.

The second set of workshops will be exclusively for LGBTIQ+ people and their (chosen) families. Participants will present their pictures, select a few to be included in the Rainbow Book, and write a story describing the connection between the image and the recommendation. This visual component will serve as a powerful form of expression, helping HCPs and policymakers perceive the world from the perspective of LGBTIQ+ people and their (chosen) families.^54^ Several celebration events will be held to present the book, reunite participants, share photographs and stories obtained via the photovoice activity and provide feedback on the recommendations co-developed. These events will also mark the conclusion of the project and could become an important stepping stone for further collaborations. Outreach activities will be co-designed and coordinated in collaboration with the NAB, the regional boards and participants, ensuring that the project’s outcomes have a lasting impact and that the community is recognised and celebrated for its contributions.

## WP1—mixed-methods research with LGBTIQ+ communities

### Research questions

We here outline our methodological approaches to achieve objectives 1 and 2 of the project:

1. To investigate shared and distinct needs in PEOLC among LGBTIQ+ subpopulations.
2. To identify how LGBTIQ+ people and their (chosen) families access palliative care and prepare for end-of-life care.

### WP1.1—secondary data analysis of existing datasets

It then entails a secondary data analysis of pertinent national surveys from Switzerland. In WP1, we will analyse recently collected data from the Swiss Health Survey (ESS), which is a nationwide survey conducted every 5 years by the Federal Statistical Office (FOPH) of Switzerland. It aims to monitor the health status, behaviours and healthcare utilisation of the Swiss population aged 15 and older. The survey collects comprehensive data on various aspects of health, including physical and mental health, chronic diseases, healthcare access and usage, health behaviours and social determinants of health. The data gathered from the Swiss Health Survey (ESS) helps to identify health trends, evaluate the effectiveness of health policies and programmes, and inform public health planning and policy making in Switzerland.

The last wave of the survey was conducted from 17 January 2022 to 22 December 2022, and included 21 930 participants. Since the last wave in 2022, a new module on gender identity and sexual orientation was introduced, asking the following questions: ‘Here are some questions regarding your sex, gender identity and sexual orientation. What sex were you assigned at birth? (male/female); Do you identify as … (men/women/non-binary people (neither exclusively men nor exclusively women)/other/I don’t know); Do you consider yourself as … (heterosexual/gay/lesbian/bisexual/other/I don’t know); People differ in terms of sexual attraction to others. Which best describes your feelings? Do you feel … (attracted only to women/mostly attracted to women/equally attracted to men and women/mostly attracted to men/attracted only to men/I’m not sure)’. Analysing the ESS data, including these new questions, represents a unique first opportunity to have a form of representative data regarding LGBTIQ+ people in Switzerland and their attitudes and behaviours concerning health. This first step will allow us to compare the results we will find with other data collected during this project.

In a second step, WP1.1 will analyse data from the Survey of Health, Ageing and Retirement in Europe (SHARE), a multidisciplinary, cross-national and longitudinal general-purpose research data infrastructure that gathers microdata on the life circumstances of people aged 50+ from 28 European participating countries.^55^ The biennial SHARE surveys focus on the dynamic relationship between health, socioeconomic status and social networks, collecting detailed data on demographics, family relationships, physical and mental health (including biomarkers and functional tests), cognitive function, healthcare use, employment, pensions, social support, income, assets, housing, activities, future expectations and information on deceased respondents through interviews with their relatives. Switzerland has participated in all nine SHARE rounds since 2004, with interviews conducted face-to-face in German, French and Italian. Additionally, country-specific self-completion questionnaires are administered, varying by wave.

The Swiss SHARE team has already conducted two specialised end-of-life questionnaires: in 2015 (wave 6) and 2019/2020 (wave 8). Another wave is being conducted in 2024/2025 (wave 10). Although the SHARE study did not directly assess gender and only asked participants whether they consider themselves male or female until wave 9, wave 10 of SHARE Switzerland includes the same questions on gender identity as the ESS. This novel addition resulted from the collaboration between the TRUST-PALL team and the Swiss SHARE Team. Moreover, in addition to the new questions, the richness of the longitudinal data will allow us to construct a gender variable for the previous waves. By applying robust methodologies, such as the Stanford Gender-Related Variables for Health Research tool,^56^ we will use SHARE-related variables associated with gender norms, gender-related traits and gender relations (eg, caregiver strain, work strain, independence, risk-taking, emotional intelligence, social support and discrimination) to analyse attitudes and behaviours toward PEOLC in Switzerland. This approach will enable us to identify patterns and trends specific to older adults and to further compare them with the new questions from Wave 10 and the results we will gather from other data collected during this project.

Finally, WP1.1 will analyse recently collected data from the Swiss LGBTIQ+ Panel, which is a longitudinal study led by two project authors (LE and TH) of this project, assessing the situation of LGBTIQ+ people in Switzerland.^57^ The panel has been conducted annually since 2019. The annual surveys have been completed by over 2500 LGBTIQ+ people and 500 cisgender heterosexual endosex (hereafter, cis-heterosexual) people from all Swiss cantons, making it Switzerland’s largest and most diverse longitudinal LGBTIQ+ study. A central aspect is its inclusion of various subgroups within the LGBTIQ+ community, as well as cis-heterosexual participants, representing diverse social group memberships and regions of Switzerland. These diverse participant characteristics allow for a fundamentally intersectional approach, with special attention to people’s sexual identities and a focus on the experiences of intersex as well as trans, non-binary and other gender-diverse people.

In the Swiss LGBTIQ+ Panel, sexual orientation, gender identity, transgender status and intersex status are measured with the following questions: ‘What is your sexual orientation? (heterosexual; bisexual; pansexual; homosexual; asexual; other, namely:); Which of the following best describes your gender identity? (man; woman; non-binary; another gender, namely:); Are you a trans person? (yes, a trans man; yes, a trans woman; yes, a trans person with a non-binary gender identity; yes, trans person. I use the following term to describe myself:; no); Are you an intersex person? (yes, an intersex man; yes, an intersex woman; yes, an intersex person with a non-binary gender identity; yes, an intersex person, I use the following term for myself:; no)’. The Swiss LGBTIQ+ Panel includes questions on other key demographics (eg, age, (dis-)ability, region, level of education, migratory status) and minority stress items (eg, coming out, discrimination, support, internalised stigma, well-being, health).

Further, the most recent wave of the Swiss LGBTIQ+ Panel survey includes open-ended questions regarding specific challenges faced by older LGBTIQ+ people, as well as experiences with the medical context and end-of-life care (eg, ‘Which particular challenges or needs do you think should be prioritised for older LGBTIQ+ people? We welcome all personal experiences or insights you may have on this topic. For example, in relation to families of origin or chosen families, friends, specific health challenges, HCPs, assisted living facilities, society at large and any other areas.; What specific actions, resources or changes do you think would make the biggest difference in improving the lives of older LGBTIQ+ people?’). As the PAR approach has a limited number of participants that can be included, the open-ended questions would once again allow for the inclusion of the LGBTIQ+ community’s perspective on a larger scale. W1.1 will thus first start by analysing existing modules covering discrimination, support systems, health issues and health-promoting and health-damaging behaviours. The open-ended responses will be translated into English and analysed using text processing packages in R or new AI-specific tools to identify general and recurrent topics. These data will provide us with key insights into specific challenges and needs of the LGBTIQ+ community. As a part of WP0.4, three workshops will be held to identify key elements to explore in the following steps. Data analyses in WP1.1 will primarily be descriptive and exploratory. We will compute prevalence estimates, cross-tabulations and multivariable regression models (eg, ordinary least squares and probit) to examine associations between sociodemographic characteristics, sexual orientation, gender identity and indicators of health, care access and end-of-life preparedness. The three data sources, the SHS, SHARE and the Swiss LGBTIQ+ Panel, will be analysed separately using methods appropriate to each dataset, and findings will later be triangulated to identify convergent and divergent patterns. Given the novelty of these data, the analyses are not hypothesis-driven but aim to generate empirically grounded insights that will inform the development of hypotheses and instruments in WP1.2 and WP1.3.

### WP1.2—qualitative data collection and analysis

#### Research questions

WP1.2 consists of a qualitative component addressing the first two aims of the project by employing multiple qualitative research designs and techniques.

#### Design and study procedures

WP1.2 employs a qualitative exploratory design. Interviews will be conducted with the support of a semi-structured interview guide, informed by existing knowledge synthesis and feedback from the PAR approach. The type of semi-structured interview will be tailored according to the population.

The interviews will include a social mapping component where participants identify and place their closest connections on a sociogram. This approach follows established qualitative social network mapping methods, which use visual sociograms to explore interpersonal relationships, communication and support structures in health contexts.^58 59^ Questions will explore the nature of these relationships, their communication with these contacts about health, healthcare and end-of-life care, as well as end-of-life or advanced care planning, healthcare proxies and advance directives. Interviews will also explore participants’ experiences with healthcare systems. For those with life-limiting illnesses, the interview will explore their illness trajectory, healthcare experiences and the roles of their social connections, particularly in relation to trust. (Chosen) families will be asked about the deceased person’s illness, the healthcare received and their experience with PEOLC planning. These three groups will be recruited via multiple strategies.

#### Population and inclusion criteria

It will target three groups: (1) older LGBTIQ+ people (participants who are part of the LGBTIQ+ community and are over 65 years of age); (2) LGBTIQ+ people with life-limiting illnesses (people who are part of the LGBTIQ+ community and have been diagnosed with a life-limiting illness); and (3) chosen family members of deceased LGBTIQ+ people. The third group will include chosen family members—those who were significant to the deceased LGBTIQ+ person, for example, a partner, living companion, member of the family of origin, person designated as a healthcare proxy or emergency contact, or someone who was notably present in the deceased person’s life. All participants must be able to participate in an interview in French, German, Italian or English.

#### Sample size

WP1.2 will generate data through approximately 30 semi-structured qualitative interviews; 10 in the German-speaking part, 10 in the French-speaking part and 10 in the Italian-speaking part. These interviews will be spread over the three participant groups, such that there are approximately three people from each linguistic region in each of the three participant groups.

#### Recruitment

We will build on the PAR approach, and advertise through mailing lists and newsletters from LGBTIQ+ associations and other stakeholders. The Swiss LGBTIQ+ Panel will also help promote the study by advertising it through their social media channels. Another approach to recruiting participants will involve collaboration with HCPs working in palliative care units at participating hospitals, institutions and other relevant settings. In locations where it is permitted, HCPs may wear Pride badges to signify their support, creating opportunities to engage in conversations with patients and their family members when appropriate. Additionally, flyers and other informational materials will be made available in hospitals, checkpoint clinics and any other locations that permit their distribution.

#### Data analysis

To analyse the data collected in WP1.2, we will use reflexive thematic analysis,^60 61^ which involves familiarisation, generating codes, constructing themes, revising and defining themes, and producing a written report.^62^ Transcripts from interviews with older LGBTIQ+ people, those with life-limiting illnesses and chosen family members will be coded to identify key themes. Social mapping components will be analysed to understand participants’ support networks and trust levels. We will compare findings across groups and subgroups to uncover shared and unique experiences. The analysis will be validated through member-checking and stakeholder feedback, ensuring the results are accurate and representative. Member-checking and stakeholder feedback will be conducted within the participatory framework of WP0, primarily through the interactive data analysis workshops.

### WP1.3—quantitative data collection and analysis

#### Research questions

WP1.3 consists of a quantitative component addressing the first two aims of the project by employing quantitative research designs and techniques.

#### Design and study procedures

To answer these aims, we will develop a new module on PEOLC for the 2026 wave of the Swiss LGBTIQ+ Panel survey. This process will be informed by our earlier knowledge synthesis (WP1.1), qualitative research findings (WP1.2) and participatory co-design activities (WP0.4). The questionnaire will include both closed-ended and open-ended questions on a range of topics: ageing-related needs of LGBTIQ+ people; the role of biological and/or chosen families in care; experiences of inclusion or exclusion in healthcare and community settings; and suggestions for improving access to and quality of PEOLC. By incorporating both quantitative and qualitative items, the questionnaire will allow us to capture patterns and variations in experiences while also foregrounding individual voices. Before fielding the full survey, the questionnaire will be pretested by members of the multilingual research team and by participants involved in the PAR process to ensure clarity, cultural and linguistic appropriateness, and comprehensibility across all four national languages.

#### Population and inclusion criteria

The primary population targeted by this study includes individuals who self-identify as part of the LGBTIQ+ community and reside in Switzerland. Participants must be aged 14 or older and able to complete the questionnaire in one of the four national languages (German, French, Italian) or English. The study aims to include individuals from a wide range of backgrounds, ensuring representation across regions, gender identities, sexual orientations and sociodemographic profiles.

#### Sample size

The Swiss LGBTIQ+ Panel currently has over 7000 active email contacts. Based on previous waves and our planned outreach strategies, we anticipate collecting responses from at least 2500 participants. This sample size will allow for robust statistical comparisons across key subgroups and ensure the generalisability of findings within the Swiss LGBTIQ+ population.

#### Recruitment

Recruitment will be conducted through multiple channels. All previous participants of the Swiss LGBTIQ+ Panel will be re-contacted via the existing email list. In addition, the research team will collaborate with major LGBTIQ+ associations (such as QueerAltern, LOS, TGNS, PinkCross, InterAction, Dialogai and Voqueer) to promote the study through newsletters, websites and social media platforms. Participation will also be encouraged at public events such as Pride marches and the PinkApple Film Festival. These efforts, combined with the trust established by the Swiss LGBTIQ+ Panel in previous years, are expected to boost participation and ensure a wide reach across different segments of the community.

#### Data analysis

Quantitative data will be analysed using R statistical software. Descriptive statistics will be calculated to summarise key variables. To explore subgroup differences, we will conduct χ^2^ tests, t-tests and analyses of variance. Latent profile analysis will be employed to identify clusters of participants with similar needs and experiences. We will also perform multivariable regression analyses (OLS and probit models) to examine associations between individual characteristics (such as age, sex, gender, sexual orientation, migratory status, socioeconomic position and disability) and outcomes related to care access, experiences and preferences. These analyses will provide a detailed and intersectional understanding of how different dimensions of identity shape end-of-life care needs within the LGBTIQ+ population.

## WP2—mixed-method research with HCPs

WP2 will collect data from HCPs related to their experiences providing PEOLC to LGBTIQ+ patients and (chosen) families in Switzerland. This WP employs an *exploratory sequential design*,^63^ meaning that an initial qualitative phase will inform a subsequent quantitative phase of data collection. WP2 specifically focuses on project objectives 3 and 4:

1. Exploring HCPs’ perceptions and understanding of LGBTIQ+ people in PEOLC.
2. Identifying how HCPs deliver PEOLC to LGBTIQ+ people.

### WP2.1—qualitative component

#### Research questions

How do healthcare professionals experience the provision of PEOLC to LGBTIQ+ people in PEOLC in Switzerland? How do professional roles, level of training, institutional support, ethical and legal considerations, workplace culture, personal beliefs and cultural humility influence healthcare professionals’ perceptions and understandings of LGBTIQ+ people’s PEOLC needs?

#### Design and study procedures

We will generate data through semi-structured qualitative interviews with HCPs providing PEOLC throughout Switzerland. Interviews will be conducted with the support of a semi-structured interview guide and include questions about professionals’ experiences providing PEOLC care to LGBTIQ+ patients, LGBTIQ+ patients’ specific needs, and perceived competencies and level of comfort providing care to LGBTIQ+ patients. In line with the PAR approach, the interview guide and preliminary results will be discussed with patient representatives and HCPs involved in the project’s participative workshop discussions. The purpose of these discussions will be to iteratively inform the study tools and to discuss emerging topics that are potentially relevant to the research that merit more in-depth investigation.

#### Population

##### Sample size

We aim to conduct approximately 30 semi-structured qualitative interviews throughout Switzerland, with 10 interviews in each main language region (German-, French- and Italian-speaking). Although we expect to attain data saturation^64^ with this sample size, our sampling strategy will allow us the flexibility to adjust the number of interviews until we have enough data to answer the research questions. Furthermore, we may pursue additional interviews, depending on PAR discussions with the project’s participative governance structure.

##### Inclusion criteria

- Belongs to at least one of the following professional categories: physician, nurse, psychologist, social worker, chaplain/spiritual advisor. These are the HCPs who compose the majority of the palliative care workforce in Switzerland.
- Provides specialised PEOLC.
- Has at least 12 months of professional experience in settings where PEOLC is provided.
- Is aware of having provided palliative care to LGBTIQ+ people.
- Can respond to qualitative interview questions in German, French, Italian or English.

##### Recruitment

We will recruit participants in healthcare institutions by sharing study flyers with hospitals, clinics and units where PEOLC services are offered, as well as via invitations in professional society mailing lists, newsletters and forums. We will also use snowball sampling by asking participants to suggest other potentially eligible study participants.

### Data analysis

Interviews will be audio-recorded and transcribed verbatim into the original language of the utterance. Accounting for Switzerland’s language and cultural differences between its regions, interviews will be conducted by researchers with advanced language skills and who are trained in qualitative research methods. Data will be analysed with the use of the qualitative data analysis software, MAXQDA.^65^ Qualitative multilingual data will be addressed through an iterative process of conceptual equivalence of meaning and language.^66^

Qualitative data from healthcare professional interviews in WP2 will be analysed using *reflexive thematic analysis*, as described by Braun and Clarke.^60 61^ Reflexive thematic analysis is an interpretive, iterative approach that actively generates themes from data through researcher engagement, reflexivity and subjectivity. This method employs systematic coding, interpretative identification of themes organised around core concepts, and continuous theme refinement. Through ongoing reflective practice and contextual awareness, it allows us to align with the participatory and inclusive principles of PAR, enabling meaningful interpretation of healthcare professionals’ perceptions and practices related to LGBTIQ+ inclusivity in PEOLC.

### WP2.2—quantitative component

#### Research questions

What are the perceived and experienced barriers and facilitators to providing inclusive PEOLC to LGBTIQ+ people, and how prevalent are these perceptions among HCPs? To what extent do healthcare providers perceive a need for additional training in inclusive PEOLC, and in what areas?

#### Design and study procedures

We will design an anonymised online survey for healthcare professionals working in Switzerland, with the support of the REDCap software. The survey will be distributed and available in German, French, Italian and English. The survey will contain questions related to HCPs’ experiences in providing care to LGBTIQ+ people, interactions with LGBTIQ+ people’s (chosen) families, recognition of specific care needs, level of personal comfort in providing care to LGBTIQ+ people, and their desire or need for additional training. For HCPs desiring additional training, the survey will ask participants to specify their preferred training content and format. Findings from the qualitative interviews conducted in WP2.1 will directly inform the development of the WP2.2 survey, ensuring that the questionnaire captures key themes, challenges and training needs identified by healthcare professionals during the qualitative phase. Before launching the full survey, the questionnaire will be pretested by multilingual members of the research team as well as by HCPs participating in the PAR process, to ensure linguistic accuracy, clarity and relevance across Switzerland’s linguistic regions and professional categories.

#### Population

##### Sample size

The target population will be heterogeneous and stratified. To allow for significant comparison analysis for subgroup (profession and language region) and multivariate regression, we aim to recruit a minimum of 300–400 participants, stratified for language and profession, which should include around 65 participants per professional group. These sample sizes are based on feasibility considerations and prior experience from similar national surveys, ensuring sufficient precision for subgroup and regression analyses.

##### Inclusion criteria

- Belongs to at least one of the following professional categories: physician, nurse, psychologist, social worker or chaplain/spiritual advisor. These are the HCPs who compose the majority of the palliative care workforce in Switzerland.
- Has at least 12 months of professional experience in settings where PEOLC is provided.
- Can respond to survey questions in German, French, Italian or English.

##### Recruitment

The survey will be advertised via professional organisations such as the Swiss Society for Palliative Care (palliative.ch), the Swiss Association of Nurses (ASI/SBK), the Swiss Society of Geriatrics (SFGG-SPSG), the Swiss Association for Gerontological Nursing and the Swiss Society of General Internal Medicine (SGAIM) to access the views of general practitioners. PEOLC providers will be invited to participate in the survey through professional society emails and/or newsletters, with a brief study description and a link to initiate survey participation. Every participant will also be invited to share the survey with their colleagues, so we also foresee a parallel snowball sampling.

### Data analysis

To analyse the survey data, we will use R and STATA software to conduct statistical analyses. These will include descriptive statistics and comparisons across subgroups (eg, professional category, linguistic region, experience levels), followed by multivariable regressions to explore associations between HCPs’ characteristics and their comfort levels, recognition of LGBTIQ+ care needs and interest in additional training. This approach will help identify key factors influencing providers’ experiences and training needs in delivering PEOLC to LGBTIQ+ people.

### Data integration within and between the WPs

To ensure a comprehensive understanding of data from WP1.2 and WP1.3, we will use a convergent mixed-methods design, giving equal weight to qualitative and quantitative findings. A translational analysis will be conducted to triangulate the data, identifying areas of convergence and divergence between the qualitative and quantitative patterns, following established mixed-methods integration frameworks.^63 67^ This approach allows us to combine the different perspectives captured through the national panel survey with the depth of individual experiences explored in the interviews. It is particularly valuable to examine how structural and social factors influence both perceived and lived experiences of PEOLC among diverse LGBTIQ+ subgroups.

In line with WP2’s *exploratory sequential design*,^63^ an initial qualitative phase will inform the subsequent quantitative data collection. Similar to WP1, this mixed-methods approach will more clearly articulate findings between methods and highlight where each dataset adds unique insights. The results will provide a nuanced understanding of HCPs’ experiences and challenges and inform the development of inclusive and effective care practices.

Data integration within and between the project’s WPs will additionally benefit from the application of the Dodd *et al data integration framework* for mixed-methods studies, following its five suggested steps: data reduction, data transformation, data comparison, integration and credibility check.^67^ This framework will allow for enhancing coherence across the diverse data types through all WPs, enabling intersectional and layered insights about perceptions and needs both from patients and HCPs. This will provide an effective integration of participants’ voices into the Rainbow Book, contributing towards evidence-informed, community-led recommendations.

## Ethics and dissemination

This study has received relevant ethical approvals from the Swiss Ethics Committees (WP0: CER-VD: Req-2025-00145; P1.3: PhF Ethics Committee of the University of Zurich: 24.12.17, WP1.2 (to be submitted), WP2.1: Req-2023-01327, WP2.2: to be submitted as an amendment to Req-2023-01327). Findings will be disseminated through peer-reviewed publications focusing on the distinct needs of LGBTIQ+ subpopulations in PEOLC, HCP experiences and perceptions, and the PAR methodology itself. In addition to the Rainbow Book, a collaboratively developed guide outlining recommended best practices for LGBTIQ+ inclusive care in PEOLC contexts, the project will produce policy briefs and outreach materials, including brochures and infographics tailored to various stakeholders. Workshops and webinars will be conducted to introduce the Rainbow Book. Ongoing communication with stakeholders will also occur through newsletters, social media, conference presentations and community events, ensuring broad accessibility to research outcomes.
