# Supplementary material for: Mixed-methods, participatory action research study exploring palliative and end-of-life care for LGBTIQ+ people in Switzerland: a protocol for the TRUST-PALL study
Source: BMJ Open. 2026 Jan 7;16(1):e106641. doi: 10.1136/bmjopen-2025-106641 (PMC12781992; doi:10.1136/bmjopen-2025-106641)
Supplement: Supplementary file 1 [file bmjopen-2025-106641.reviewer_comments.pdf]

PEER REVIEW HISTORY

BMJ Open publishes all reviews undertaken for accepted manuscripts. Reviewers are asked to complete a checklist review form and are provided with free text boxes to elaborate on their assessment. These free text comments are reproduced below.

ARTICLE DETAILS

Title (Provisional)

A mixed methods, participatory action research study exploring palliative and end-of-life care for LGBTIQ+ people in Switzerland: a protocol for the TRUST-PALL study

Authors

Deml, Michael J.; Meier, Clément; Letras, Catarina; Canova, Nina; Martinelli, Paolo; Jones, Laura; Colussi, Gaé; Larkin, Philip J; Eisner, Léïla; Hässler, Tabea; Bosisio, Francesca; Gamondi, Claudia

---

VERSION 1 - REVIEW

**Reviewer**	**1**
**Name**	**Hashim, Jumana**
**Affiliation**	**National University of Singapore, School of Medicine**
**Date**	**01-Sep-2025**
**COI**	**None**

The authors have designed a thorough and thoughtful study that incorporates various methodologies. This is an important topic, one that is complex in its exploration, translation, and eventual implementation. The authors can benefit from considering some of the points provided in the attached document.

Reviewer comments:

1. Introduction can benefit from being more concise – For example, paragraphs 5-7 can be shortened to make the points clearer and direct.
2. The PAR principles listed are useful; however, some elaboration as to why these were chosen (as opposed to the other), and potentially a citation on where the authors got these principles from will be useful.
3. How were the principles practised, tested, and disseminated within the project?
4. If the steps in WP0 were adapted from previous literature, citations would be helpful in reference to the demonstrated efficacy. Within the step of WP0, multiple methodologies are listed – for example, the prioritisation activities in WP0.3 and WP0.5 – these approaches themselves have protocols that the authors may benefit from articulating. Another example is WP0.4 – codesigning of the data collection materials – there are many approaches to doing this; elaborating this would be expected in a protocol.

5. The Rainbow Book is referred to in WP0.6, but some introduction to it, and its wider purpose will be useful – is this a term widely used, or is this novel to the study?
6. WP1.1 – Please provide details on the data analysis plan. Is there any hypothesis that the authors are looking to test?
7. In WP1.1, three data sources will be used – will they be triangulated and/or combined?
8. Is WP1.2 in parallel or sequential to WP1.1. Authors can consider whether the findings from WP1.1 may inform their interview guides and study design for WP1.2.
9. Authors can consider including samples of the interview guide as supplementary materials – the interview guide can benefit from organising the various topics that are outlined on pages 15, lines 13-19.
10. There is an additional social mapping component – more information and/or citation to the methodology will be required.
11. Given the multi-level stratification, the methodology only recruits about 3 participants per sub-group. Although this is a qualitative exploration, the number may be relatively low, and the authors may want to explore data saturation at one of the stratification levels (instead of all) as an indicator of sufficient data collection.
12. Will the recruitment strategy be the same for all 3 types of participants?
13. How will member-checking and stakeholder feedback be conducted? I suspect this is where there is merging with WP0 – authors will benefit from mentioning this clearly.
14. For WP1.3 – will the newly developed survey questions be piloted? Is there any hypothesis to be tested? What is the statistical exploration that a 2500 participant sample size is supposed to answer?
15. How do the different recruitment strategies plan for minimising any biases?
16. Is there a reason why Braun and Clarke are referenced in WP2.1 but not in WP1.2 for the reflexive thematic analysis? How will these two qualitative data analyses differ?
17. Since WP2.2 is sequential to WP2.1 and the findings will inform the survey design, the authors may want to mention this on Page 19 under 'design and study procedures'
18. How are these sample sizes calculated? Providing some references or justification will be required.
19. References for the data triangulation on page 20, lines 24-25 will be required.
20. Authors can benefit from discussing some methodology limitations and how they have planned to mitigate these limitations in their design. Highlighting the strengths of this comprehensive study design will also be beneficial.

VERSION 1 - AUTHOR RESPONSE

Reviewer 1:

The authors have designed a thorough and thoughtful study that incorporates various methodologies. This is an important topic, one that is complex in its exploration, translation, and eventual implementation. The authors can benefit from considering some of the points provided in the attached document.

We sincerely thank the reviewer for their encouraging feedback on the importance and design of our study. We appreciate the recognition of the complexity of the topic and the value of our methodological approach. We have carefully reviewed the points provided in the attached document and have addressed them in the revised manuscript, as detailed in our responses below.

1. Introduction can benefit from being more concise – For example, paragraphs 5-7 can be shortened to make the points clearer and direct.

We thank the reviewer for this suggestion and fully appreciate the importance of clarity and conciseness in the Introduction. However, we have chosen to retain the current level of detail in paragraphs 5–7, as these sections provide the essential epidemiological, contextual, and conceptual background necessary to situate the study within both the Swiss and international palliative care landscape. In particular, these paragraphs, i) bridge general end-of-life research with the specific focus on LGBTIQ+ populations, clarifying why existing evidence cannot be directly extrapolated to this group, ii) provide necessary national context regarding mortality patterns and decision-making frameworks in Switzerland, which justify the study's focus and the choice of a participatory, mixed-methods design, and iii) highlight key evidence gaps for example, the lack of Swiss data on inclusive palliative care and healthcare professionals' preparedness, directly motivating the project's objectives. We believe that condensing these sections would risk losing critical nuance about (a) why LGBTIQ+ people's experiences at the end of life are uniquely shaped by systemic and social determinants, and (b) how this justifies a participatory action research approach in the Swiss multilingual context.

2. The PAR principles listed are useful; however, some elaboration as to why these were chosen (as opposed to the other), and potentially a citation on where the authors got these principles from will be useful.

We thank the reviewer for this helpful suggestion. We have now clarified why the four selected PAR principles, collaboration and participation, co-construction of knowledge, reflexivity and positionality, and social justice and emancipatory goals, were chosen and added supporting references already cited in the manuscript (Cornwall & Jewkes, 1995 [43]; Cargo & Mercer, 2008 [47]). Specifically, we added the following explanatory sentence at the end of the paragraph introducing the PAR principles:

"*These four principles were selected as they represent the core foundations of participatory action research in health, emphasizing co-learning, empowerment, and social transformation [43,47]. They align closely with the TRUST-PALL study's goal to co-create inclusive and contextually grounded knowledge with LGBTIQ+ communities in sensitive palliative and end-of-life contexts.*"

3. How were the principles practised, tested, and disseminated within the project?

We thank the reviewer for this insightful comment. The practical implementation of the PAR principles is already detailed throughout section "WP0 - Participatory Action Research." Specifically, the six steps (WP0.1–WP0.6) describe how the principles are practiced, for instance, through the creation of a participative governance structure (WP0.1), ongoing relationship-building and trust (WP0.2), and the co-design of data collection tools and analysis (WP0.4–WP0.5). The principles are tested and iteratively refined through participant feedback loops, validation workshops, and member-checking processes described in WP0.2 and WP0.5, which ensure reflexivity and responsiveness to participant input. Finally, the dissemination of these principles is explicitly integrated into WP0.6 through the co-creation of the Rainbow Book and the organization of public workshops and community events, which translate the co-produced knowledge into actionable and sustained outputs.

4. If the steps in WP0 were adapted from previous literature, citations would be helpful in reference to the demonstrated efficacy. Within the step of WP0, multiple methodologies are listed – for example, the prioritisation activities in WP0.3 and WP0.5 – these approaches themselves have protocols that the authors may benefit from articulating. Another example is WP0.4 – codesigning of the data collection materials – there are many approaches to doing this; elaborating this would be expected in a protocol.

We thank the reviewer for this helpful observation. The six steps of WP0 were not directly adapted from a single existing framework but were developed collaboratively by the research team, drawing upon established PAR and participatory health research literature already cited in the manuscript (e.g., Cornwall & Jewkes, 1995 [43]; Cargo & Mercer, 2008 [47]; Eklund Karlsson et al., 2019 [48]; Goedhart et al., 2021 [51]). These references demonstrate the proven efficacy of participatory and co-design approaches in health research and community engagement. Regarding the specific methodologies mentioned (e.g., prioritization activities and co-design of instruments), we have intentionally kept the level of description consistent with BMJ Open's expectations for a study protocol. Each technique mentioned, such as the Agreement-&-Certainty Matrix [50], Nominal Group Technique [52], and Photovoice [53], already includes an appropriate citation and a brief description of its function. Detailed procedural information (e.g., step-by-step facilitation or coding templates) will be presented in subsequent methodological papers following data collection, to avoid redundancy and maintain the protocol's clarity. We therefore believe that the current manuscript appropriately balances methodological transparency with conciseness while ensuring that every participatory activity listed in WP0 is grounded in referenced and validated approaches.

5. The Rainbow Book is referred to in WP0.6, but some introduction to it, and its wider purpose will be useful – is this a term widely used, or is this novel to the study?

We thank the reviewer for this helpful comment. The Rainbow Book is a novel dissemination and knowledge-translation output developed specifically for the TRUST-PALL study. The term is not widely used in existing literature; rather, it was deliberately chosen to symbolize inclusivity, visibility, and diversity, values central to both the LGBTIQ+ community and the participatory philosophy underpinning the project. To address this comment, we have added a short explanatory sentence introducing the Rainbow Book and its purpose. The new text reads:

"*The 'Rainbow Book' is a novel output of the TRUST-PALL project. It symbolizes inclusivity and diversity and will serve as a co-created best-practice guide translating project findings into accessible recommendations for LGBTIQ+ people, their (chosen) families, healthcare professionals, and policymakers.*"

6. WP1.1 – Please provide details on the data analysis plan. Is there any hypothesis that the authors are looking to test?

We thank the reviewer for this valuable comment. WP1.1 involves secondary analyses of three existing national datasets (Swiss Health Survey, SHARE, and the Swiss LGBTIQ+ Panel). The analytic approach is primarily exploratory and descriptive, as the main objective is to identify patterns and gaps in existing data concerning LGBTIQ+ people's health and end-of-life experiences in Switzerland. To clarify this point, we have added a short paragraph specifying the analysis plan and the absence of formal hypothesis testing at this stage. The new text reads:

"*Data analyses in WP1.1 will primarily be descriptive and exploratory. We will compute prevalence estimates, cross-tabulations, and multivariable regression models (e.g., OLS and probit) to examine associations between socio-demographic characteristics, sexual orientation, gender identity, and indicators of health, care access, and end-of-life preparedness. Given the novelty of these data, the analyses are not hypothesis-driven but aim to generate empirically grounded insights that will inform the development of hypotheses and instruments in WP1.2 and WP1.3.*"

7. In WP1.1, three data sources will be used – will they be triangulated and/or combined?

Thank you for this important question. The three datasets in WP1.1, the Swiss Health Survey (ESS), SHARE, and the Swiss LGBTIQ+ Panel, will not be statistically combined due to

different sampling frames, target populations, and measurement instruments. Instead, they will be analyzed separately and triangulated analytically/conceptually to identify converging and contrasting patterns across datasets, thereby strengthening inference and informing subsequent components of the study.

8. Is WP1.2 in parallel or sequential to WP1.1. Authors can consider whether the findings from WP1.1 may inform their interview guides and study design for WP1.2.

We thank the reviewer for this thoughtful observation. WP1.1 and WP1.2 are designed to be sequential and complementary rather than strictly parallel. Findings from the secondary analyses in WP1.1 will directly inform the development of the semi-structured interview guides and sampling strategy for WP1.2. Specifically, patterns and gaps identified in the existing datasets will be used to refine interview topics and ensure that under-represented perspectives are explored in greater depth during the qualitative phase. This sequencing is consistent with the project's iterative participatory design, where each stage builds on the insights generated in the preceding one.

9. Authors can consider including samples of the interview guide as supplementary materials – the interview guide can benefit from organising the various topics that are outlined on pages 15, lines 13-19.

We thank the reviewer for this helpful suggestion. The interview guides are currently under development as part of the PAR process. Their content is being co-designed and refined collaboratively with LGBTIQ+ community members, their (chosen) families, and healthcare providers during the preparatory workshops. Because these guides are an evolving outcome of the PAR process, they are not yet finalized and therefore cannot be included as supplementary materials at this stage. Once finalized, they will be made publicly available alongside future publications reporting on the qualitative findings.

10. There is an additional social mapping component – more information and/or citation to the methodology will be required.

We thank the reviewer for this helpful observation. We agree that a citation supporting the use of the social mapping component would strengthen the methodological clarity. We have now added a reference to established applications of social network mapping in qualitative health research. Specifically, we have included the following sentence in WP1.2:

"*This approach follows established qualitative social network mapping methods, which use visual sociograms to explore interpersonal relationships, communication, and support structures in health contexts [58,59].*

11. Given the multi-level stratification, the methodology only recruits about 3 participants per sub-group. Although this is a qualitative exploration, the number may be relatively low, and the authors may want to explore data saturation at one of the stratification levels (instead of all) as an indicator of sufficient data collection.

We thank the reviewer for this thoughtful comment. We fully acknowledge that, given the linguistic and subgroup stratification, the planned number of interviews per category is relatively small. However, our design aims to ensure broad representational diversity across Switzerland's three main linguistic regions while maintaining feasibility and depth of inquiry for each subgroup. In qualitative research, data saturation is typically assessed within thematic domains rather than across all stratification levels. We therefore plan to monitor saturation at the thematic level, that is, when no new concepts or experiences emerge within each main participant category (older LGBTIQ+ people, LGBTIQ+ people with life-limiting illnesses, and chosen family members), rather than strictly by linguistic or demographic subgroup.

12. Will the recruitment strategy be the same for all 3 types of participants?

We thank the reviewer for this pertinent question. While the overall recruitment strategy will follow the same participatory and community-based approach across all three participant types, the specific recruitment pathways will be adapted to each group. For older LGBTIQ+ people and those with life-limiting illnesses, recruitment will primarily occur through LGBTIQ+ associations, community networks, and collaborating healthcare providers. For chosen family members of deceased LGBTIQ+ people, recruitment will rely more on snowball sampling and community referrals facilitated by trusted associations, given the sensitivity of this group. This flexible, tailored strategy ensures inclusivity while respecting participants' contexts and levels of vulnerability.

13. How will member-checking and stakeholder feedback be conducted? I suspect this is where there is merging with WP0 – authors will benefit from mentioning this clearly.

We thank the reviewer for this helpful comment. Member-checking and stakeholder feedback will indeed be conducted through the participatory workshops and feedback loops described in WP0, particularly WP0.5 ("Interactive data analysis"). After initial coding and thematic synthesis, participants and stakeholders will be invited to discuss and validate preliminary findings in structured workshops organized across linguistic regions. This process allows participants to confirm the accuracy and relevance of interpretations and to co-refine emerging themes. To clarify this, we have included the following sentence:

"*Member-checking and stakeholder feedback will be conducted within the participatory framework of WP0, primarily through the interactive data analysis workshops.*"

14. For WP1.3 – will the newly developed survey questions be piloted? Is there any hypothesis to be tested? What is the statistical exploration that a 2500 participant sample size is supposed to answer?

We thank the reviewer for this important and detailed comment. The new survey module to be developed in WP1.3 will undergo internal pretesting and community consultation in line with standard procedures of the Swiss LGBTIQ+ Panel. Each new module is reviewed by the research team and partner organizations and is pre-tested for clarity, linguistic equivalence, and acceptability in German, French, and Italian before full implementation in the annual online wave. WP1.3 is primarily exploratory rather than hypothesis driven. Its purpose is to identify patterns and subgroup differences in palliative and end-of-life care experiences, needs, and preferences among LGBTIQ+ people in Switzerland. The expected sample of approximately 2,500 participants ensures sufficient representation across gender identities, sexual orientations, and linguistic regions, enabling robust descriptive analyses, subgroup comparisons, and multivariable regression models (e.g., OLS, probit, and latent profile analysis).

15. How do the different recruitment strategies plan for minimising any biases?

We thank the reviewer for this thoughtful question. We acknowledge that engaging diverse LGBTIQ+ populations presents inherent recruitment challenges and potential selection biases. To minimize these, the study employs several complementary strategies. Recruitment will occur through a broad range of channels, including national and regional LGBTIQ+ associations, healthcare institutions, community networks, and social media, ensuring access to individuals with varying levels of visibility and engagement. In addition, collaboration with community organizations and the involvement of "bridge persons" (members of both the research and LGBTIQ+ communities) will help build trust and reach individuals who are less connected to formal networks. For healthcare providers, recruitment through multiple professional associations and snowballing will similarly broaden representation across professional roles,

regions, and care settings. This combination of multi-channel outreach, participatory engagement, and reflexive monitoring aims to reduce selection bias and ensure that a wide diversity of voices and experiences is represented in the study.

16. Is there a reason why Braun and Clarke are referenced in WP2.1 but not in WP1.2 for the reflexive thematic analysis? How will these two qualitative data analyses differ?

We thank the reviewer for this pertinent observation. Both WP1.2 and WP2.1 will use reflexive thematic analysis following the approach developed by Braun and Clarke. The omission of their reference in WP1.2 was unintentional. We have now added the appropriate citation to ensure consistency across work packages.

17. Since WP2.2 is sequential to WP2.1 and the findings will inform the survey design, the authors may want to mention this on Page 19 under 'design and study procedures'

We thank the reviewer for this helpful observation. We agree that clarifying the sequential link between WP2.1 and WP2.2 strengthens the methodological transparency of the protocol. We have therefore added a sentence to the "Design and study procedures" subsection of WP2.2 stating that:

"*Findings from the qualitative interviews conducted in WP2.1 will directly inform the development of the WP2.2 survey, ensuring that the questionnaire captures key themes, challenges, and training needs identified by healthcare professionals during the qualitative phase.*"

18. How are these sample sizes calculated? Providing some references or justification will be required.

We thank the reviewer for this helpful comment. As this is an exploratory mixed-methods study, sample sizes were determined based on methodological guidance and feasibility rather than formal power calculations. For the qualitative components (WP1.2 and WP2.1), the target of approximately 30 interviews per work package aligns with recommendations for reaching thematic saturation in diverse, multi-site qualitative research (Saunders et al., 2018 [64]). For the quantitative components (WP1.3 and WP2.2), sample sizes follow established precedents: around 2,500 participants from the Swiss LGBTIQ+ Panel in each annual wave and 300–400 healthcare professionals recruited through national associations, providing adequate precision for subgroup analyses. We have added the following clarifying sentence in WP2.2:

"*These sample sizes are based on feasibility considerations and prior experience from similar national surveys, ensuring sufficient precision for subgroup and regression analyses.*"

19. References for the data triangulation on page 20, lines 24-25 will be required.

We thank the reviewer for this helpful observation. We agree that providing references for the data triangulation approach strengthens methodological transparency. We have therefore added citations to established frameworks for data integration and triangulation in mixed-methods research, which are already referenced elsewhere in the manuscript. Specifically, we now cite Dodd et al. (2020 [67]) and Creswell & Plano Clark (2017 [63]) to support the approach described on page 20, lines 24–25. The revised sentence reads:

"A translational analysis will be conducted to triangulate the data, identifying areas of convergence and divergence between qualitative and quantitative patterns, following established mixed-methods integration frameworks [63,67]."

20. Authors can benefit from discussing some methodology limitations and how they have planned to mitigate these limitations in their design. Highlighting the strengths of this comprehensive study design will also be beneficial.

We thank the reviewer for this valuable suggestion. We have therefore added a short paragraph at the end of the Strengths and limitations of this study section:

"Coordinating participatory processes across multiple linguistic regions and integrating qualitative and quantitative findings may present methodological challenges; these are mitigated through multilingual engagement strategies and a structured mixed-methods integration framework. Despite these challenges, the study's participatory, multi-level, and interdisciplinary design represents a major strength, enabling the co-production of actionable, context-specific evidence for inclusive palliative and end-of-life care."
